# Exosomes released from educated mesenchymal stem cells accelerate cutaneous wound healing via promoting angiogenesis

**DOI:** 10.1111/cpr.12830

**Published:** 2020-07-01

**Authors:** Xinyu Qiu, Jin Liu, Chenxi Zheng, Yuting Su, Lili Bao, Bin Zhu, Siying Liu, Lulu Wang, Xiao Wang, Yirong Wang, Wanmin Zhao, Jun Zhou, Zhihong Deng, Shiyu Liu, Yan Jin

**Affiliations:** ^1^ State Key Laboratory of Military Stomatology & National Clinical Research Center for Oral Diseases & Shaanxi International Joint Research Center for Oral Diseases Center for Tissue Engineering School of Stomatology The Fourth Military Medical University Xi’an China; ^2^ State Key Laboratory of Military Stomatology & National Clinical Research Center for Oral Diseases & Shaanxi Key Laboratory of Stomatology Department of Prosthodontics School of Stomatology The Fourth Military Medical University Xi'an China; ^3^ Xi’an Institute of Tissue Engineering and Regenerative Medicine Xi’an China; ^4^ Department of Aerospace The Fourth Military Medical University Xi’an China; ^5^ Department of Stomatology The General Hospital of Tibet Military Region Lhasa China; ^6^ State Key Laboratory of Military Stomatology & National Clinical Research Center for Oral Diseases & Shaanxi Clinical Research Center for Oral Diseases Department of Orthodontics School of Stomatology The Fourth Military Medical University Xi’an China; ^7^ State Key Laboratory of Military Stomatology & National Clinical Research Center for Oral Diseases and Shaanxi Clinical Research Center for Oral Diseases Department of Pediatric Dentistry School of Stomatology The Fourth Military Medical University Xi'an China; ^8^ State Key Laboratory of Military Stomatology & National Clinical Research Center for Oral Diseases & Shaanxi Key Laboratory of Oral Diseases Department of Operative Dentistry and Endodontics School of Stomatology The Fourth Military Medical University Xi'an China

**Keywords:** angiogenesis, exosome, mesenchymal stem cell, regenerative medicine, wound healing

## Abstract

**Objectives:**

Skin serves as the major interface between the external environment and body which is liable to many kinds of injuries. Mesenchymal stem cell (MSC) therapy has been widely used and became a promising strategy. Pre‐treatment with chemical agents, hypoxia or gene modifications can partially protect MSCs against injury, and the pre‐treated MSCs show the improved differentiation, homing capacity, survival and paracrine effects regard to attenuating injury. The aim of this study was to investigate whether the exosomes from the educated MSCs contribute to accelerate wound healing process.

**Materials and methods:**

We extracted the exosomes from the two educated MSCs and utilized them in the cutaneous wound healing model. The pro‐angiogenetic effect of exosomes on endothelial cells was also investigated.

**Results:**

We firstly found that MSCs pre‐treated by exosomes from neonatal serum significantly improved their biological functions and the effect of therapy. Moreover, we extracted the exosomes from the educated MSCs and utilized them to treat the cutaneous wound model directly. We found that the released exosomes from MSCs which educated by neonatal serum before had the more outstanding performance in therapeutic effect. Mechanistically, we revealed that the recipient endothelial cells (ECs) were targeted and the exosomes promoted their functions to enhance angiogenesis via regulating AKT/eNOS pathway.

**Conclusions:**

Our findings unravelled the positive effect of the upgraded exosomes from the educated MSCs as a promising cell‐free therapeutic strategy for cutaneous wound healing.

## 1 INTRODUCTION

Skin is the largest organ of body which serves as the first defence line to the external environment. However, it makes the skin tissue liable to many kinds of injuries like incision, burn and infection.^1^ Impaired cutaneous wound healing may become life‐threatening and is a major public health issue worldwide.^2^ To date, various strategies have been explored, and mesenchymal stem cell (MSC) therapy shows a great potential.^3^ More and more studies have shown that MSC therapy could promote cutaneous wound healing and reorganized the structure of skin.^4^, ^5^ Meanwhile, pre‐treatment of MSCs could enhance the function and efficacy of therapy, and many studies focus on how to optimize MSCs before their applications.^6^, ^7^, ^8^ However, many studies have shown that only part of transplanted MSCs can eventually survive and incorporate into the host tissues.^9^, ^10^, ^11^, ^12^, ^13^ Therefore, improving the functions of MSC before transplantation is a good choice to ensure the effect of therapy. As we all known, young MSCs have a more powerful therapeutic effect, but whether it is affected by the cell microenvironment in serum is still eye‐catching.

Several studies have pointed out a key class of mediators that function between MSCs and target cells: exosomes, a population of extracellular vesicles,^14^ were shown as the important regulators of cell functions.^15^, ^16^ Exosomes are abundant in circulation system and could influence target cells by transmitting factors including proteins, mRNAs, microRNAs and so on.^17^, ^18^ The components of exosomes are varied in different conditions, which are likely to be involved in various pathophysiology environments in the body. For example, the exosomes derived from aged cells are different from that derived from normal cells.^19^ The specific changes in the components of exosomes have been adopted in disease diagnosis and cell‐free therapy.^16^, ^20^ Moreover, local exosomes secreted by stem cells in hypothalamus have been revealed to affect ageing and locally delivering the exosomes secreted by young hypothalamic stem cells slows down the ageing of mice.^21^ Meanwhile, the exosomes from the pre‐treated MSCs emerged a therapeutic effect on wound healing, which reminded us using the upgraded exosomes for treatment and regulating tissue regeneration might be a good approach.

Thus, in this study, we selected the cutaneous wound healing model to observe the effect of the exosomes which from serum of neonatal and adult mice on MSCs, and the potential therapeutic effect of exosomes derived from the educated MSCs. In our present study, we firstly compared the differences between the exosomes from serum of neonatal and adult mice. Then, we investigated the biological effect of two exosomes on MSCs and compared the therapeutic ability in cutaneous wound repair between these two educated MSCs. We found that the MSCs educated by exosomes from neonatal serum had a better therapeutic effect on wound healing process. Moreover, we extracted the exosomes from two educated MSCs and utilized them to treat the cutaneous wound directly to compare the therapeutic efficacy. We found that two released exosomes both had the therapeutic effect, and the exosomes released from MSCs which educated by neonatal serum before had the more outstanding performance. Mechanistically, we revealed that the recipient endothelial cells were targeted, and the exosomes promoted their functions to enhance angiogenesis via regulating AKT/eNOS pathway. In conclusion, we verified the positive effect of exosomes from neonatal serum on MSCs and provided the upgraded exosomes from educated MSCs as a promising cell‐free therapeutic strategy for cutaneous wound healing.

## 2 MATERIALS AND METHODS

### 2.1 Animals

Animal experiments were approved by the Institutional Animal Care and Use Committee of the Fourth Military Medical University (No.2018066). Wild‐type (WT) neonatal and adult C57BL/6J mice were purchased from the Laboratory Animal Center of the Fourth Military Medical University (FMMU). The mice were kept under specific pathogen‐free conditions (25°C, 12‐h light/dark cycles and 50% humidity) with free access to food and water.

### 2.2 Cell isolation and culture

Bone marrow mesenchymal stem cells (BMMSCs) were isolated from the femurs and tibias of adult C57BL/6J mice. In brief, MSCs were drawn out by flushing from bones with basal culture medium containing α‐MEM medium (Gibco, 12571063), 20% exosome‐free FBS (Sijiqing, 13011‐8611), 2 mM L‐glutamine (Invitrogen, 25030‐081), 100 U/mL penicillin and 100 U/mL streptomycin (Invitrogen, 15140‐122). Single‐cell suspension was equally seeded in dishes and initially maintained in an atmosphere of 5% CO_2_ at 37°C. The human umbilical vein endothelial cells (HUVECs) were purchased from ATCC (CRL‐1730) and cultured in special medium (ScienCell, A1001) according to the instructions.

### 2.3 Isolation and characterization of exosomes

The neonatal mice (5‐7 g) that were 14 days old and the adult mice (20‐22 g) that were 12 weeks old were used. Firstly, the mice underwent anaesthesia by an intra‐peritoneal injection of pentobarbitone sodium (40 mg/kg). Then, the whole blood was collected from heart and approximately 150‐200 μL whole blood could be collected from each neonatal mouse, and 1‐1.3 mL whole blood could be collected from each adult mouse. The serum was separated from whole blood and combined for further exosomes isolation. Approximately 40 μL serum could be separated from 100 μL whole blood. Exosomes were isolated by using the optimized protocol. The serum was mixed with an equal volume of phosphate‐buffered saline (PBS) and centrifuged at 2000 *g* for 30 minutes; then, the supernatant was centrifuged at 12 000 *g* for 45 minutes. The supernatant was transferred into a fresh tube, filtered with a 0.22 μm filter and pelleted by ultracentrifugation (Beckman Optima L‐100 XP, Beckman Coulter) at 110 000 *g* for 120 minutes. Exosome pellets were washed in a large volume of PBS and recovered by centrifugation at 110 000 *g* for 70 minutes (Figure S1).

Firstly, the primary MSCs were cultured in α‐MEM with 20% exosome‐free FBS and all conditions of culturing MSCs were uniform before pre‐treatment. Then, the second passage of MSCs was pre‐treated with exosomes (20 μg/mL) from neonatal or adult serum for 24 hours under basic medium without FBS. After pre‐treatment, the fresh FBS‐free medium was changed and MSCs were cultured for another 48 hours. Finally, the conditioned medium was collected to isolate exosomes by ultracentrifugation. Briefly, cell culture supernatant was centrifuged at 2000 *g* for 10 minutes. Next, the supernatant was collected and centrifuged at 10 000 *g* for 30 minutes. The final supernatant is then ultracentrifuged at 100 000 *g* for 70 minutes. The pellet was washed in a large volume of PBS to remove contamination of proteins and ultracentrifuged at 100 000 *g* for 70 minutes once more (Figure S2).

The collected exosomes were resuspended in PBS and quantified by BCA assay (TIANGEN, PA115) before using and stored at −80°C for further study if necessary. Approximately 25 μg exosomes could be isolated from 1 mL serum. In all experiments, neonatal and adult serum exosomes were used with the same and uniform concentrations. Exosomes were fixed in 4% paraformaldehyde (Sigma‐Aldrich, 16005), washed and loaded onto copper grids. After washing, exosomes were post‐fixed in 2% glutaraldehyde for 2 minutes, washed and contrasted in 2% phosphotungstic acid for 5 minutes. Samples were washed and dried, and images were obtained with an electron microscope (HITACHI, H7500). The size distribution was measured by dynamic light scattering (DLS) analysis by using Zetasizer Nano ZSE. The proteins expression of purified exosomes for CD9, CD63, CD81 and TSG101 was detected by Western blot. The primary antibodies involved in this study include CD9 (1:400, Abcam, ab92726), CD63 (1:800, Abcam, ab217345), CD81 (1:200, Abcam, ab109201) and TSG101 (1:200, Abcam, ab125011).

### 2.4 Flow cytometric analysis

Cell phenotypes of cultured BMMSCs at P2 were detected by flow cytometric analysis. For identification of the expression of mesenchymal stem cell surface markers, cells were harvested and washed by PBS. Then, the single‐cell suspension was incubated with fluorescein isothiocyanate (FITC)‐conjugated mouse anti‐CD11b, anti‐CD29, anti‐CD45, anti‐Sca‐1 and allophycocyanin (APC)‐conjugated anti‐CD34, anti‐CD90, anti‐CD105 and anti‐CD146 (all from eBioscience), respectively. Related conjugated IgG was used as control. Finally, cells were washed twice in PBS and subjected to flow cytometric analysis with Beckman Coulter CytoFLEX (Beckman Coulter).

### 2.5 Colony‐forming unit assays

To assess the colony‐forming efficiency of MSCs, single‐cell suspensions with α‐MEM containing 20% FBS were seeded in 5 cm diameter culture dishes (Corning, 430166) at a density of 1 × 10^2^ cells per well and cultured at 37°C in a humidified atmosphere containing 5% CO_2_. The medium was refreshed every other day. After culturing for 5 days, the dishes were rinsed with PBS and the cells were fixed by 4% paraformaldehyde (Sigma‐Aldrich, 16005). The cells were stained with 0.2% crystal violet (Sigma‐Aldrich, C6158), washed with distilled water and dried for evaluation under the inverted optical microscope (Leica, M205FA).

### 2.6 Osteogenic differentiation assay

Mesenchymal stem cells were seeded in six‐well plates at a density of 5 × 10^5^ cells per well. When cells reached 100% confluence, the basal medium was changed into osteogenic induction medium: α‐MEM containing 20% FBS, 1% penicillin/streptomycin, 5 mM β‐glycerophosphate, 50 μg/mL ascorbic acid and 10 nM dexamethasone (all from Sigma‐Aldrich). The medium was refreshed every other day. For alkaline phosphatase (ALP) staining, after 10 days the medium was discarded, and the samples were washed with PBS twice and fixed with 4% paraformaldehyde (Sigma‐Aldrich). ALP staining was performed with a commercial kit (Beyotime, C3206) according to the manufacturer's protocol. Cells were cultured for 14 or 28 days, and the Alizarin red (Sigma‐Aldrich, A5533) staining was performed according to the manufacturer's instructions. Photographs were taken by an inverted optical microscope (Leica, M205FA). The 10% cetylpyridinium chloride was added for quantitative analysis, and the absorbance values were measured at 562 nm.

### 2.7 Adipogenic differentiation assay

Mesenchymal stem cells were seeded in six‐well plates at a density of 5 × 10^5^ cells per well. When cells reached 100% confluence, the basal medium was changed into adipogenic medium: α‐MEM containing 20% FBS, 0.5 mM isobutylmethylxanthine, 0.5 mM dexamethasone and 60 nM indomethacin (all from Sigma‐Aldrich). The medium was refreshed every other day. After induction for 7 or 14 days, Oil Red O (Sigma‐Aldrich, O0625) staining was performed to determine lipid droplet formation. Photographs were taken by the inverted optical microscope (Leica, M205FA). The positive area was measured by ImageJ software (NIH). The quantification was studied based on semi‐automatic plug‐ins, which followed the same operational methods.

### 2.8 Internalization of exosomes into MSCs in vitro

The MSCs were plated onto dishes and maintained at 37°C overnight. Exosomes were pre‐labelled with the PKH26 Red Fluorescent Cell Linker Kit (Sigma‐Aldrich, PKH26PCL‐1KT) according to the manufacturer's instructions and washed by PBS. PKH26‐labelled exosomes at the concentration of 0‐100 μg/mL were then co‐cultured with MSCs for 1‐12 hours. After fixed by 4% paraformaldehyde for 15 minutes at 4°C, the cells were used for cytoskeleton staining (Yeasen, 40735ES75), and the cell nuclei were counterstained with Hoechst 33342 (Sigma‐Aldrich, 14533). The fluorescence was observed by the laser scanning confocal microscope (Nikon, A1).

### 2.9 Immunofluorescence

Cells and tissue samples were fixed in 4% paraformaldehyde for 12 hours. The skin tissue samples underwent dehydration with 30% saccharose and were embedded into the optimal temperature compound (OCT) (Leica, 14020108926) and then cut into 10μm thick sections. The cells and sections underwent permeabilization with 0.05% Triton X‐100 (Sigma‐Aldrich, T8787) for 10 minutes at room temperature, blocking with 5% bovine serum albumin (BSA) (MP, 0218072801) at 37°C for 30 minutes, and incubated with the primary antibodies overnight at 4°C. Then, the cells and tissue sections were incubated with the related fluorescence secondary antibodies at room temperature for 1 hour. Finally, the nuclei were counterstained by Hoechst 33342 (Sigma‐Aldrich, 14533) for 10 minutes at room temperature. The images were obtained by a confocal microscope (Olympus, FV1000) and quantified by ImageJ software (NIH, USA). The antibodies involved in this study include the following: Ki67 (1:200, Abcam, ab15580), CD31 (1:100, R&D Systems, AF3628SP), KRT14 (1:400, Abcam, ab7800), α‐SMA (1:200, Abcam, ab5694) and Cy3‐conjugated IgG (Jackson, 111‐165‐003, 705‐165‐003 and 715‐005‐150).

### 2.10 Western blot

Cells and exosomes samples were lysed with RIPA (Beyotime, P0013) containing protease inhibitor (Roche, 04693132001). Cytosolic fractions were extracted with cytoplasmic protein extraction kit (Beyotime, P0027) according to the manufacturer's instruction. Protein quantification was performed using a BCA assay (Beyotime, P0012). The extracted proteins (20‐30 μg) were separated by SDS‐polyacrylamide gel electrophoresis (PAGE) and transferred onto polyvinylidene fluoride (PVDF) membranes (Roche, 03010040001). The membranes were blocked with 5% bovine serum albumin (MP Biomedical, 0218072801) for 2 hours at room temperature, followed by incubation with primary antibodies overnight at 4°C. After washing three times with Tris‐buffered saline‐Tween (Solarbio, T1081), the membranes were incubated with secondary antibodies for 2 hours at room temperature. Subsequently, by further washing with TBS‐T, protein bands were visualized with an enhanced chemiluminescence system using the Tanon Chemidoc Apparatus (Tanon‐Bio, 4600) and quantified by ImageJ software. The following primary antibodies were used: Runx2 (1:500, Cell Signaling Technology, 12556), ALP (1:200, R&D Systems, AF2910), PPAR‐γ (1:200, Abcam, ab209350), p‐AKT (1:1000; Cell Signaling Technology, 9271), AKT (1:1000; Abcam, ab179463), p‐eNOS (1:1000; Cell Signaling Technology, 9570), eNOS (1:1000; Cell Signaling Technology, 32027), p‐AMPK (1:1000; Cell Signaling Technology, 2535), p‐FAK (1:1000; Cell Signaling Technology, 8556), GAPDH (1:4000, CWBio, CW0100) and β‐actin (1:4000, CWBio, CW0096).

### 2.11 Skin wound healing model

The mice underwent anaesthesia by an intra‐peritoneal injection of pentobarbitone sodium (40 mg/kg). After shaving and cleaning, a full‐thickness wound (1 cm in diameter) was created on the dorsal skin. MSCs and MSC‐derived exosomes were resuspended in PBS and intra‐dermally injected around each wound with four sites, respectively, and the injection was administrated in the layer of dermis. The number of MSCs was 2 × 10^6^ (in 100 μL PBS) when they were applied to the wound site, which was calculated by the digital cell counter (Bio‐Rad). The concentration of exosomes was 100 μg (in 100 μL PBS) when they were applied to the wound site, which was measured by BCA assay (TIANGEN, PA115). In all experiments, MSCs and exosomes in different groups were used with uniform concentrations. After operation, the wound was covered by surgical dressings (3M, 9546) and the mice were kept individually to ensure that they do not bite each other. The dressing of each mouse was removed uniformly, and then, pictures were taken to observe the healing process. Particularly, we cut the film into a square shape slightly larger than the round wound in the middle of the back and ensure that the film fixed on the back of mouse is completely inaccessible. The wound area and body weight were measured on day 0 to 14 post‐surgery, and the wound healing rate was calculated by ImageJ software (NIH, USA). The mice were sacrificed, and the skin tissues were harvested for the further analysis.

### 2.12 Histological analysis

To observe the result of wound healing and collagen fibre disposition, the skin tissue samples were fixed in 4% paraformaldehyde for 24 hours and underwent dehydration with graded ethanol. Then, the samples were embedded in paraffin and cut into 4 μm thick sections. H&E staining and Masson staining were carried out by using commercial staining kits according to the manufacturer's instructions (Baso Technology, BA4025 & BA4079). Photographs were captured by a microscope (Leica, M205FA).

### 2.13 Cell migration assay

The endothelial cells (ECs) were seeded in 6‐well plates at a density of 5 × 10^5^ cells per well. When ECs reached 100% confluence, the scratch was made on the plates by using the sterile pipette tips. ECs were cultured in special medium (without FBS) and added with different exosomes at 20 μg/mL or co‐culture with the pre‐treated MSCs. Photographs were taken by an inverted microscope at 0, 12 and 24 hours and evaluated by ImageJ software (NIH, USA).

### 2.14 Tube formation assay

In vitro capillary network formation was determined by tube formation assay on Matrigel (Corning, 354248). The endothelial cells (1.5 × 10^4^ cells/mL) were seeded onto Matrigel‐coated wells of a 96‐well plate and cultured in 1% FBS‐supplemented DMEM (Gibco, 10567014) in the presence of exosomes at 20 μg/mL or the conditional medium at 100 μL. Tube formation was observed by an inverted microscope (Leica, DMi8). The number of network structures was quantified by randomly selecting five fields per well by using ImageJ software (NIH, USA).

### 2.15 Exosomes inhibition

For experiments requiring exosomal inhibition, firstly, MSCs were pre‐treated with exosomes from neonatal serum for 24 hours under basic medium without FBS. After pre‐treatment, the fresh FBS‐free medium was changed and MSCs were incubated with 20 μM GW4869 (MCE, HY‐19363) for 24 hours before exosome isolation. Finally, the pre‐treated MSCs and conditioned medium were collected for follow‐up experiments, and the effect of inhibition was measured by BCA assay.

### 2.16 Statistical analysis

Data were expressed as mean ± SD, as indicated. Comparisons between two groups were performed by Student's *t* test, and multiple group comparisons were performed by one‐way ANOVA. Bonferroni correction was used when multiple comparisons were performed. *P* values <.05 were considered statistically significant. Graphs and statistical analysis were performed by using GraphPad Prism (GraphPad Software, 7.0) and SPSS software (IBM, 19.0).

## 3 RESULTS

### 3.1 Isolation and characterization of MSCs

**FIGURE 1:**
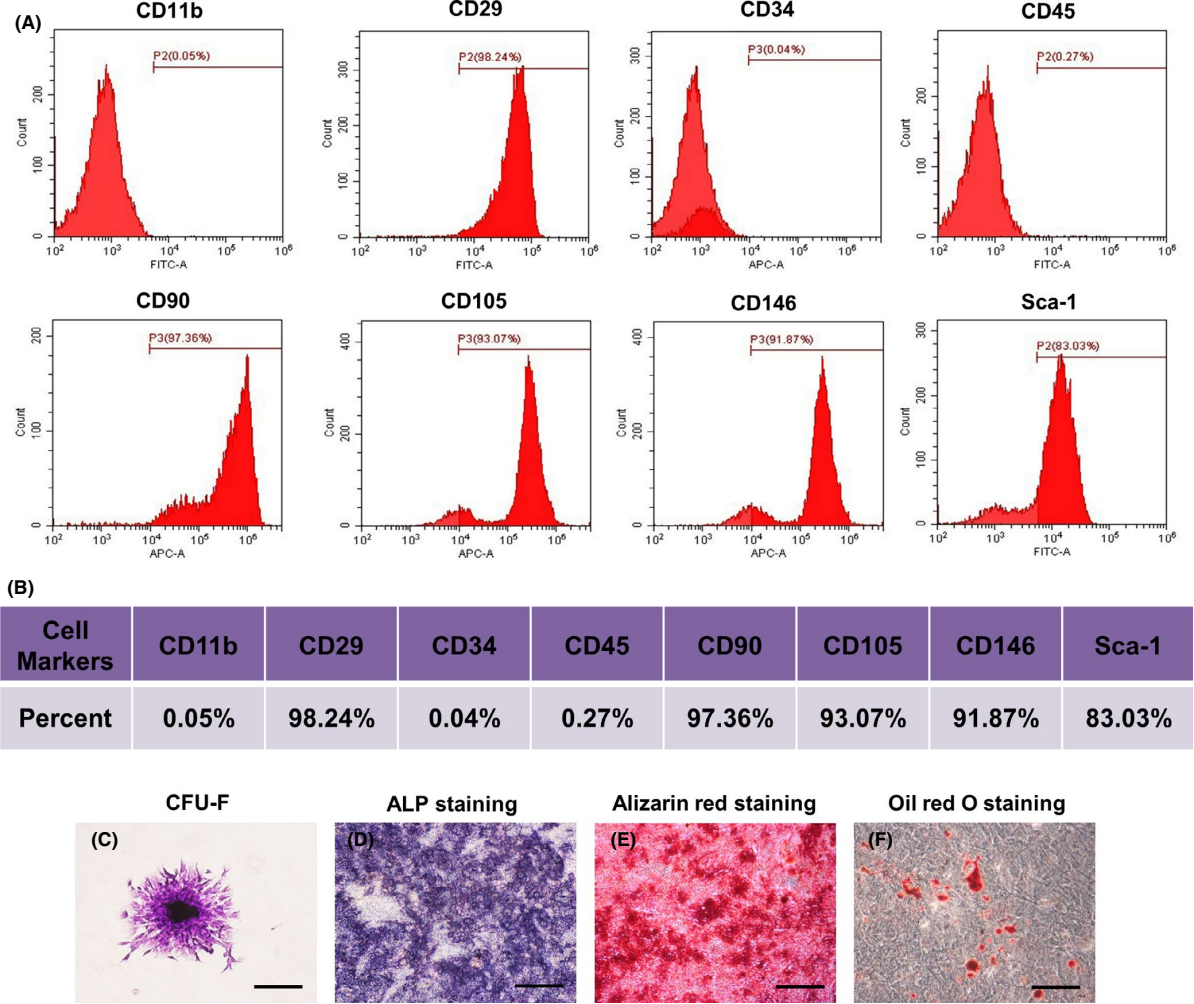
Characterization of bone marrow mesenchymal stem cells (BMMSCs). A, Flow cytometric analysis of ex vivo expanded MSCs revealed positive expression of CD29, CD90, CD105, CD146 and Sca‐1, and negative expression of CD34, CD11b and CD45. B, Representative percentage of cell surface markers for identification. C, Representative proliferation of single clone of bone marrow mesenchymal stem cells. D, MSCs seeded in plates induced with osteogenic medium for 10 days. Activity of ALP detected by ALP staining. E, MSCs cultured in osteogenic inductive conditions for 28 days, mineralized nodules found by Alizarin Red staining. F, Cultured MSCs formed Oil Red O‐positive lipid cluster following 14 days of adipogenic induction. Scale bar, 500 μm. ALP, alkaline phosphatase; APC, allophycocyanin; CFU‐F, fibroblastic colony‐forming unit; FITC, fluorescein isothiocyanate

The purified MSCs were successfully obtained from the murine bone marrow. They were propagated on a standard dish in vitro and exhibited fibroblast‐like morphology. The cells exhibited the characteristic pattern of mesenchymal stem cell surface markers, including CD29, CD90, CD105, CD146 and Sca‐1, whereas the hematopoietic markers CD34, CD11b and CD45 were negative (Figure 1A,B). When the MSCs were cultured at a low density, they formed adherent clonogenic cell clusters (Figure 1C). To investigate the differentiation potential of the MSCs, they were cultured in osteogenic differentiation medium. ALP staining was performed after 10‐day induction (Figure 1D), and mineralized nodules were stained with Alizarin Red after 28‐day induction (Figure 1E). After culturing in adipogenesis inducing medium for 14 days, the MSCs were found to form lipid droplets, as confirmed by Oil Red O staining (Figure 1F).

### 3.2 Characterization of the exosomes from serum of neonatal and adult mice

**FIGURE 2:**
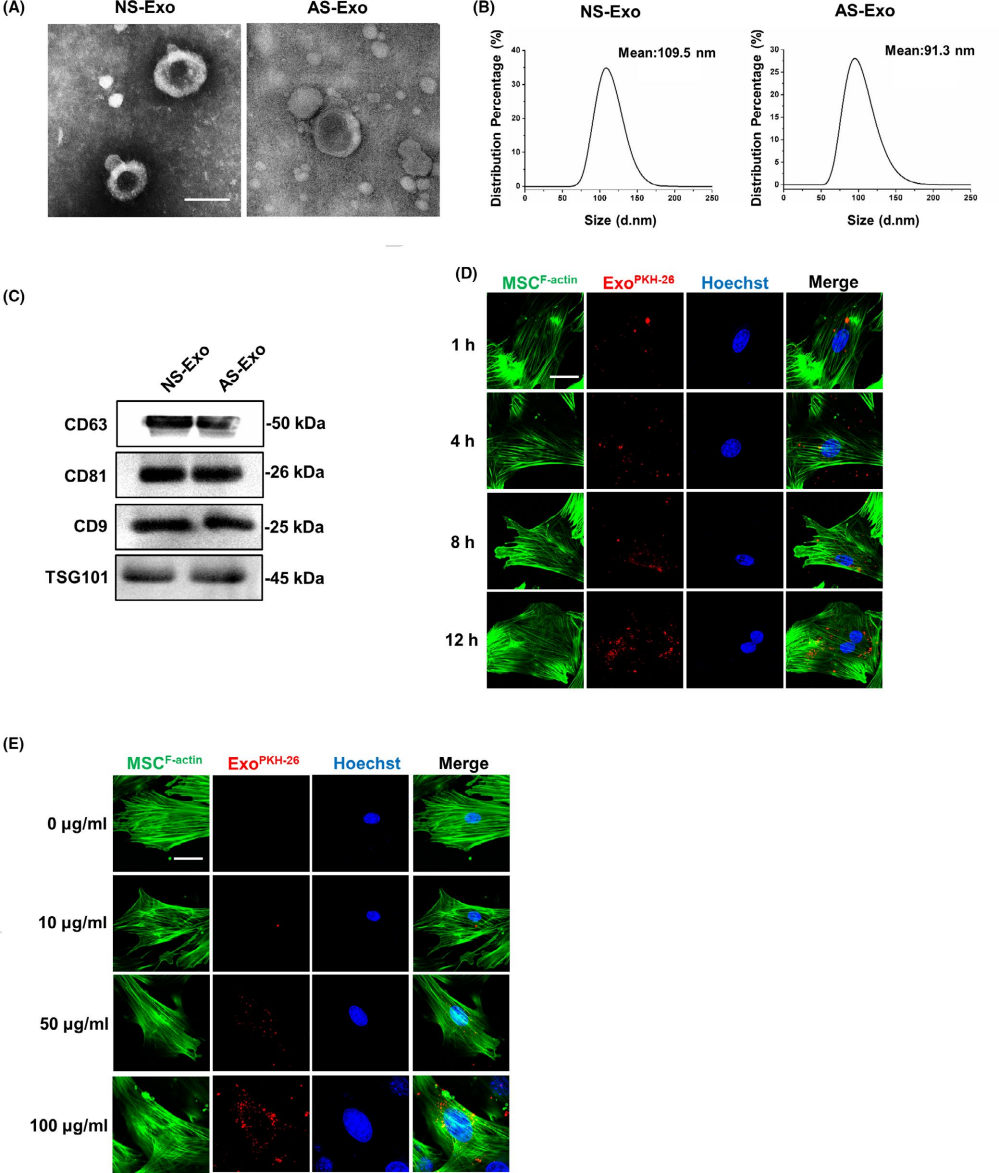
Exosome characterization in neonatal and adult serum. A, Transmission electron microscopy showing extracellular vesicles with ~100 nm diameters isolated from neonatal and adult serum. Scale bar, 100 nm. B, Size analysis revealed neonatal serum vesicles with diameters of 109.5 ± 2.1 nm and adult serum vesicles with diameters of 91.3 ± 2.3 nm. C, Total proteins extracted from nanometre vesicles probed by anti‐CD9, anti‐CD63, anti‐CD81 and anti‐TSG101 antibodies. D, The immunofluorescence images of time‐dependent uptake of exosomes by MSCs. E, The immunofluorescence images of concentration‐dependent uptake of exosomes by MSCs. n = 3 per group. Scale bar, 20 μm. AS‐Exo, adult serum exosomes; NS‐Exo, neonatal serum exosomes

As shown in the results, transmission electron microscopy revealed the vesicles from serum in the size range of ~100 nm (Figure 2A). The size distribution analysis by DLS revealed 109.5 ± 2.1 nm vesicles from neonatal serum and 91.3 ± 2.3 nm vesicles from adult serum (Figure 2B), consistent with known exosomal size.^22^ Meanwhile, the isolated exosomes from neonatal and adult serum both positive in the expression of CD9, CD63, CD81 and TSG101 were considered as the markers of classical exosomes (Figure 2C). There is no significant difference in the expression levels of these markers of exosomes between neonatal and adult serum. Moreover, to investigate the cellular uptake of exosomes, we incubated the MSCs with the PKH26‐labelled exosomes and PBS served as the negative control. It was observed that there was a significant red fluorescence signal increasing in the time‐ and concentration‐dependent way as we expected (Figure 2D,E). To verify the reliable internalization of exosomes by MSCs, confocal microscope measurements varying positions on the *z*‐axis with higher spatial resolution were also carried out (Figure S3). Therefore, these data demonstrated that little difference of morphology, size distribution and expression of surface markers from exosomes of neonatal and adult serum, and the exosomes could be uptaken by MSCs in the time‐ and concentration‐dependent way.

### 3.3 Exosomes from serum promoted the functions of MSCs

**FIGURE 3:**
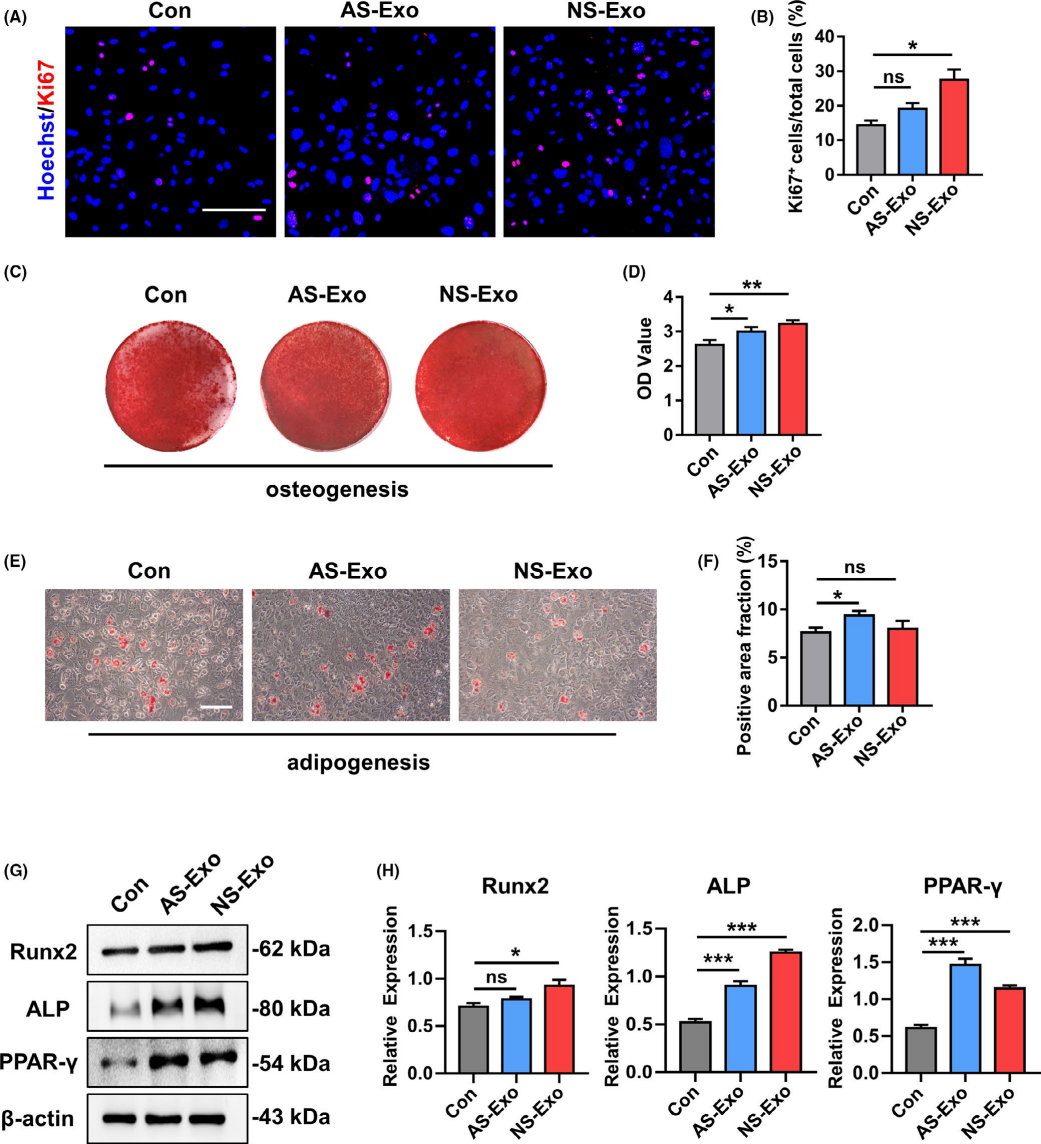
The representative images of Ki‐67 staining (A) and quantified by the positive‐stained percentage through ImageJ software (B). Scale bar, 100 μm. (C‐D) Alizarin Red staining was performed to detect mineralized nodules formed in Con, AS‐Exo and NS‐Exo 14 days after osteogenic induction and quantified with a spectrophotometer after dissolving with cetylpyridinium chloride. (E‐F) Lipid droplet formation was detected by Oil Red O staining 7 days after adipogenic induction with positive area quantified. Scale bar, 100 μm. (G‐H) The protein expression levels of Runx2, ALP and PPAR‐γ in Con, AS‐Exo and NS‐Exo groups were measured through Western blot and quantified by ImageJ software. n = 3 per group. Data are shown as mean ± SD; ns, not significant; *P < .05; **P < .01; ***P < .001. AS‐Exo, adult serum exosomes; Con, control; NS‐Exo, neonatal serum exosomes

Initially, we examined the impact of exosomes from serum of neonatal and adult mice on the functions of MSCs in vitro. We performed proliferation and lineage differentiation of MSCs assays to test the enhanced effect of exosomes from serum. MSCs proliferation was assessed by immunofluorescence staining, and the results showed that the percentage of Ki67‐positive cells significantly increased with treatment of the exosomes from serum of neonatal mice (NS‐Exo), indicating that NS‐Exo promoted the proliferation of MSCs (Figure 3A,B). Then, we detected osteogenesis of adult serum exosomes (AS‐Exo)‐treated MSCs and NS‐Exo‐treated MSCs. The Alizarin Red staining confirmed that the osteogenic differentiation was increased in NS‐Exo‐treated MSCs group (Figure 3C,D). Moreover, Oil Red O staining showed that the number of lipid droplets grew in AS‐Exo‐treated MSCs and did not change significantly in NS‐Exo‐treated MSCs (Figure 3E,F). The protein expressions of Runx2, ALP and PPAR‐γ revealed that NS‐Exo could enhance the osteogenesis of MSCs (Figure 3G,H). Taken together, these results indicated that the exosomes from neonatal serum could increase the lineage differentiation of MSCs more than from adult serum, which may enhance the functions of MSCs. Moreover, to further explore the factors in exosomes of neonatal and adult serum that making difference, we investigated the expression levels of three key proteins in serum exosomes by Western blot which play the important roles in MSC functions. The results showed that the expression levels of p‐FAK, p‐AMPK and p‐AKT in exosomes from neonatal serum were significantly higher than the exosomes from adult serum, suggesting that the exosomes from neonatal serum could have a stronger ability to regulate the biological functions of MSCs (Figure S4).

### 3.4 Educated MSCs promoted cutaneous wound healing after transplantation

**FIGURE 4:**
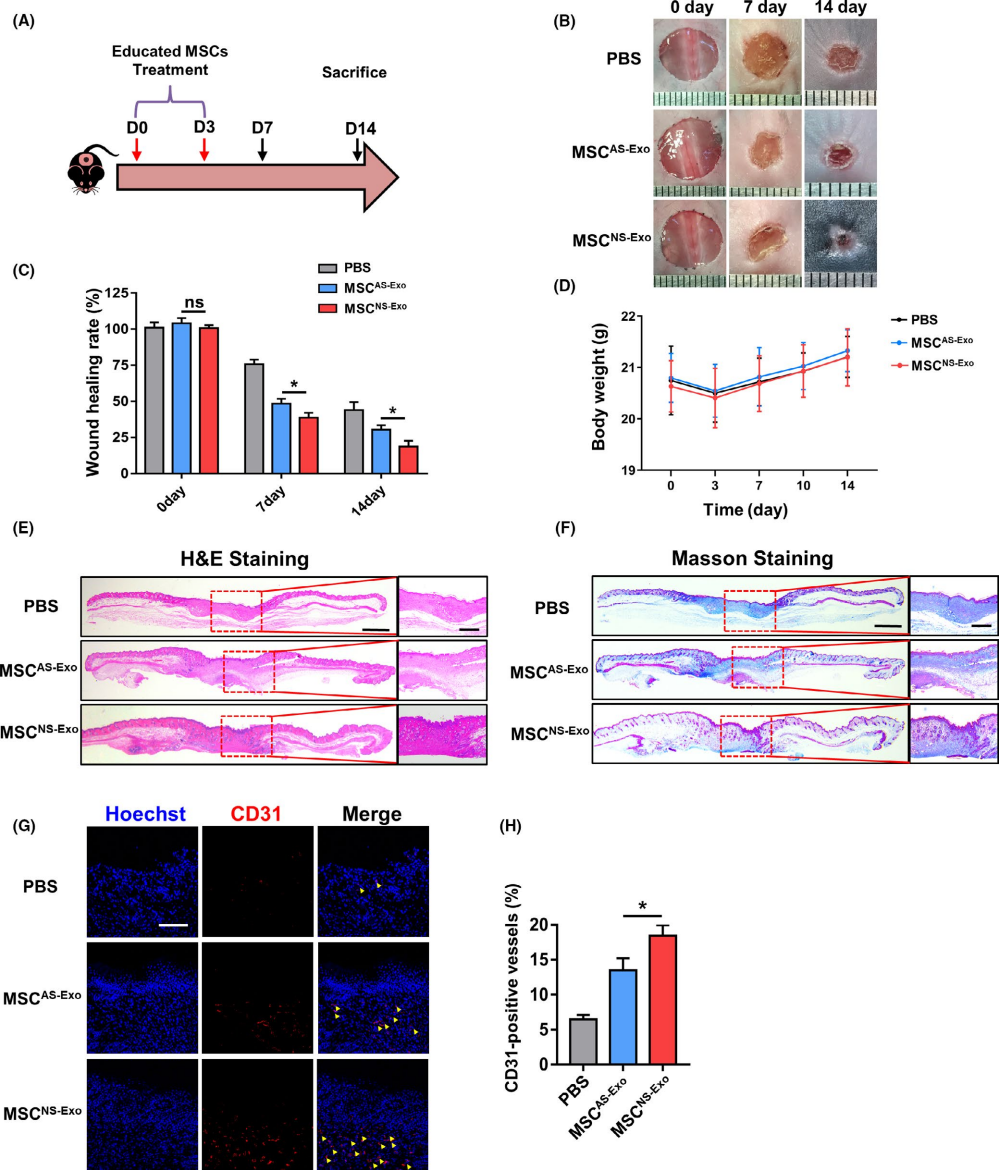
The schematic graph (A) shows the protocol of establishment and treatment for cutaneous wound healing model. (B) The representative photographs of wound healing process of different timepoints in different groups. (C) The quantification of wound healing rate. (D) The quantification of body weight in different groups. (E) Representative images of H&E staining of the skin tissue samples. Scale bar, 1 mm in low magnification images and 500 μm in high magnification images. (F) Representative images of the Masson‐trichrome staining of the skin tissue samples. Scale bar, 1 mm in low magnification images and 500 μm in high magnification images. (G‐H) Representative images and quantification of the CD31 expression in the skin tissue samples in different groups. Yellow arrows indicate the positive area. Scale bar, 100 μm. n = 3 per group. Data are shown as mean ± SD; ns, not significant; *P < .05. PBS, phosphate‐buffered saline; MSC^NS‐Exo^, MSCs educated by exosomes from neonatal serum; MSC^AS‐Exo^, MSCs educated by exosomes from adult serum

In order to verify the therapeutic efficacy of MSCs that were educated by exosomes from neonatal and adult serum, we established cutaneous wound healing model and locally employed the exogenous MSCs for treatment (Figure 4A). MSCs were resuspended in PBS and intra‐dermally injected into the wound site, and PBS was used as the negative control. Photographs of wound area were taken at three different timepoints during the wound healing process, and the wound healing rate was calculated as well (Figure 4B,C). As expected, the group of NS‐Exo educated MSCs (MSC^NS‐Exo^) healed the fastest among the three groups, while no significant differences were found in the body weight of all groups (Figure 4D). Then, we collected the skin samples at day 14 and further conducted histological analysis to assess the result of skin regeneration. As shown by H&E and Masson staining (Figure 4E,F), we observed a more integrate cutaneous structure with newly formed epithelium and appendages as well as better deposited and organized collagen in MSC^NS‐Exo^‐treated group than AS‐Exo educated MSCs (MSC^AS‐Exo^) group. In addition, we detected the expression level of CD31 by immunofluorescence staining which presents the quality angiogenesis in cutaneous wound healing. The fluorescence intensity was significantly higher in MSC^NS‐Exo^ group, which implied the better angiogenesis than in the MSC^AS‐Exo^ group (Figure 4G,H). These data demonstrated that educated MSCs accelerated the cutaneous wound healing rate and enhanced the cutaneous regenerative quality by promoting angiogenesis.

### 3.5 The educated MSC‐derived exosomes promoted cutaneous wound healing

**FIGURE 5:**
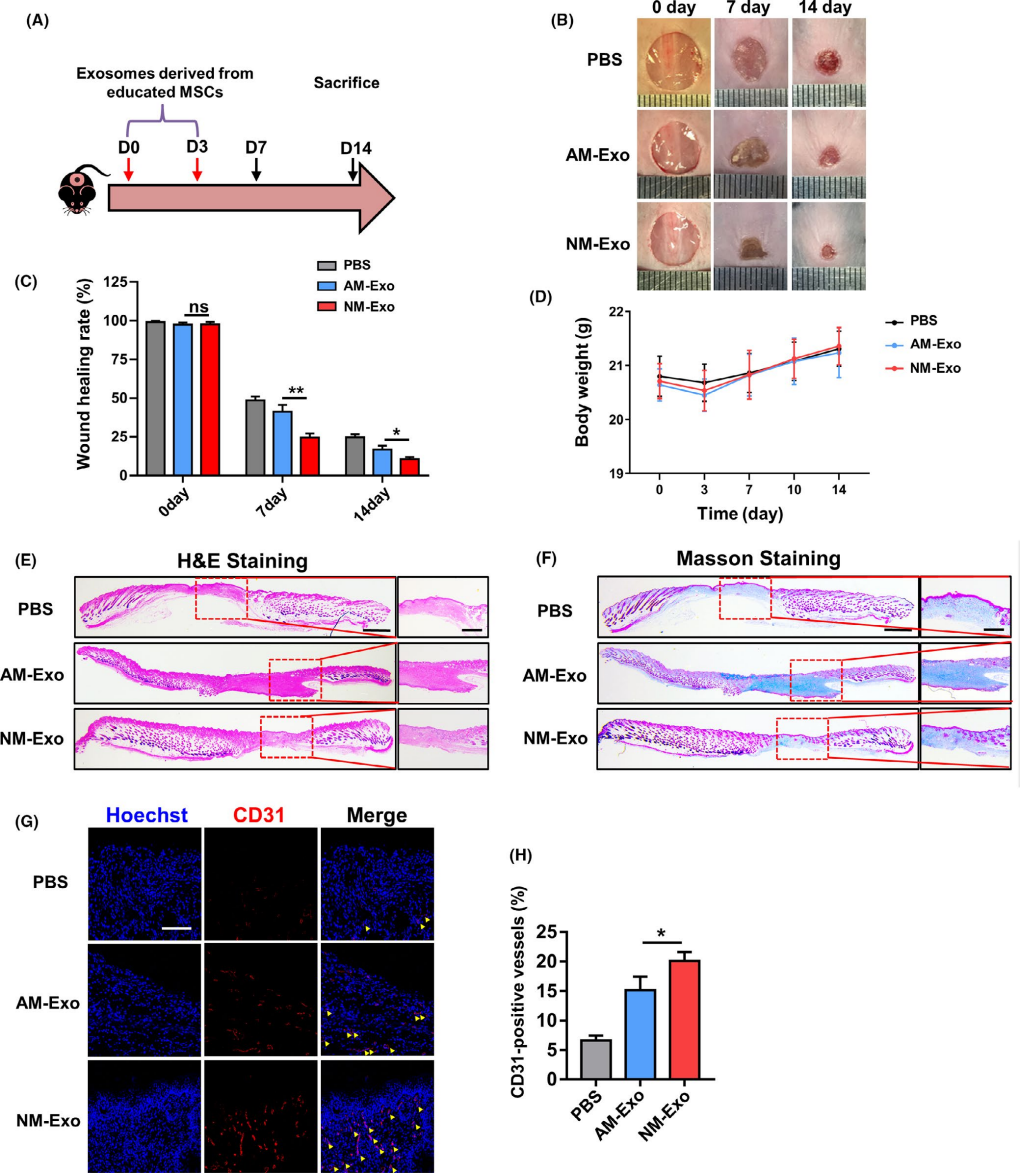
The schematic graph (A) shows the protocol of establishment and treatment for cutaneous wound healing model. (B) The representative photographs of wound healing process of different timepoints in different groups. (C) The quantification of wound healing rate. (D) The quantification of body weight in different groups. (E) Representative images of H&E staining of the skin tissue samples. Scale bar, 1 mm in low magnification images and 500 μm in high magnification images. (F) Representative images of the Masson‐trichrome staining of the skin tissue samples. Scale bar, 1 mm in low magnification images and 500 μm in high magnification images. (G‐H) Representative images and quantification of the CD31 expression in the skin tissue samples in different groups. Yellow arrows indicate the positive area. Scale bar, 100 μm. n = 3 per group. Data are shown as mean ± SD; ns, not significant; *P < .05; **P < .01. PBS, phosphate buffer saline; NM‐Exo, exosomes derived from MSC^NS‐Exo^; AM‐Exo, exosomes derived from MSC^AS‐Exo^

To further investigate the therapeutic effect of educated MSCs, we tested whether the exosomes derived from the educated MSCs could also have the same result. We locally administrated exosomes in cutaneous wound healing model by using the same methods with MSC therapy (Figure 5A). Exosomes were intra‐dermally injected into the wound site, and PBS was also used as the negative control. Photographs of wound area were taken at three different timepoints during the wound healing process, and the wound healing rate was also calculated (Figure 5B,C). As we expected, the group of exosomes derived from MSC^NS‐Exo^ (NM‐Exo) healed the fastest among the three groups, while no significant differences were found in the body weight of all groups (Figure 5D). We then collected the skin samples at day 14 and further conducted histological analysis to assess the results of skin regeneration. The H&E and Masson staining (Figure 5E,F) showed a more integrate cutaneous structure with newly formed epithelium and appendages as well as better deposited and organized collagen in NM‐Exo‐treated group than exosomes derived from MSC^AS‐Exo^ (AM‐Exo) group. In addition, re‐epithelization is an important process in cutaneous wound healing process which is mainly mediated by the migration of keratinocytes from adjacent epidermis and the proliferation of keratinocytes that feed the advancing and migrating epithelial tongue. Cytokeratin 14 (KRT14) is the important marker of keratinocytes, and the expression level of KRT14 undergoes upregulation in response to injury indicating that the keratinocytes acquire a migratory phenotype. Hence, the level of re‐epithelization can be reflected by the expression of KRT14 in keratinocytes around the wound site.^23^, ^24^, ^25^, ^26^ Therefore, we performed the immunofluorescence staining and quantitative analysis of KRT14 on the different groups. The results showed that the fluorescence intensity in the NM‐Exo group was obviously higher than the other two groups, which demonstrated that treated by NM‐Exo could promote the re‐epithelization in cutaneous wound healing (Figure S5). Meanwhile, the wound contraction is considered as part of the tissue remodelling phase of the healing process, which is mainly drove by myofibroblasts. Myofibroblasts are differentiated from fibroblasts and feature an increased expression of α‐smooth muscle actin (α‐SMA). Excessive activation of myofibroblast will lead to scar hyperplasia and fibrosis, which is undesirable in cutaneous wound healing process.^27^, ^28^ Therefore, we also performed the immunofluorescence staining of α‐SMA on the samples of different groups and found that the fluorescence intensity in the NM‐Exo group was obviously lower than the other two groups, which also demonstrated that the application of NM‐Exo could inhibit the hypertrophic scar formation in wound healing process (Figure S6). Moreover, the expression level of CD31 by immunofluorescence staining showed the better angiogenesis in NM‐Exo‐treated group than AM‐Exo (Figure 5G,H). These data demonstrated using NM‐Exo could accelerate the cutaneous wound healing and enhance the intensity of angiogenesis more powerfully.

### 3.6 Exosomes released by educated MSCs upregulated the functions of endothelial cell to promote angiogenesis

**FIGURE 6:**
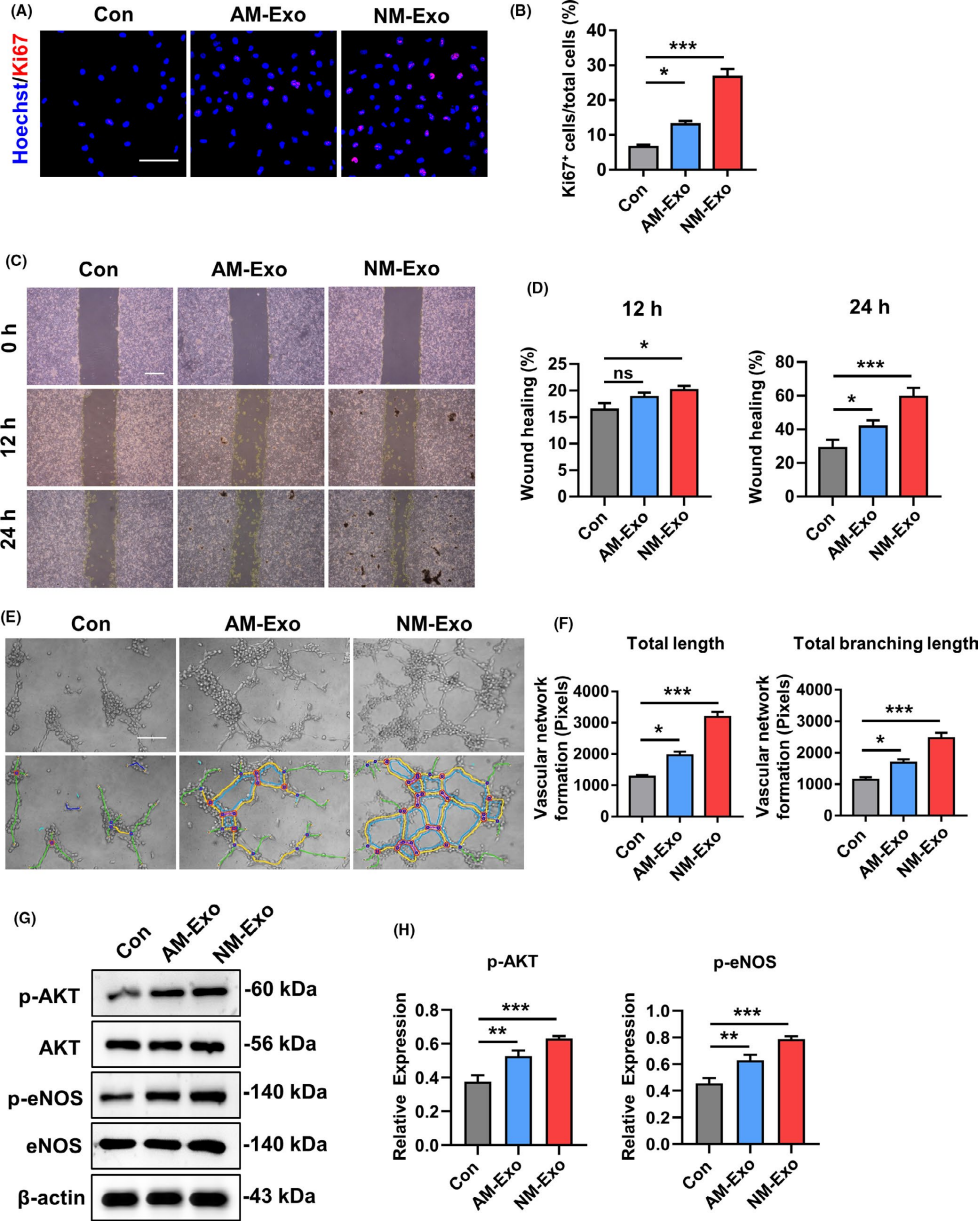
Exosomes activated the angiogenic capacity of endothelial cells in vitro. (A) Proliferation of endothelial cells was detected by Ki67 staining and quantified by the positive‐stained percentage (B). Scale bar, 100 μm. (C‐D) Representative images and quantification of scratch assay examining the migration ability of endothelial cells treated with different exosomes. Scale bar, 500 μm. (E‐F) Tube formation capacity on the Matrigel and the images were analysed by ImageJ software. Scale bar, 20 μm. (G‐H) The protein expression levels of p‐AKT, AKT, p‐eNOS and eNOS were detected by Western blot in endothelial cells which were treated with different exosomes. The results were quantified by ImageJ software. n = 3 per group. Data are shown as mean ± SD; ns, not significant; *P < .05; **P < .01; ***P < .001. Con, control; NM‐Exo, exosomes derived from MSC^NS‐Exo^; AM‐Exo, exosomes derived from MSC^AS‐Exo^

As we have shown that the exosomes derived from educated MSCs could enhance angiogenesis and improve the efficacy of cutaneous regeneration, we further examined the impact of NM‐Exo and AM‐Exo on the functions of endothelial cells (ECs) in vitro. We performed proliferation, migration and tube formation assay to test pro‐angiogenic efficacy of exosomes. EC proliferation was assessed by immunofluorescence staining of Ki‐67, and the results showed that the percentage of Ki‐67‐positive cells significantly increased with the NM‐Exo treatment, indicating that NM‐Exo had the best effect (Figure 6A,B). In addition, we evaluated the effects of different exosomes on the pro‐angiogenic capacity of ECs by migration and tube formation assay. The results demonstrated that the group of NM‐Exo had the more powerful effect on promoting the migration and tube formation of ECs (Figure 6C‐F). Meanwhile, the aim of our study was to obtain functionally improved exosomes by pre‐treating the MSCs. Therefore, to verify the key role of releasing exosomes, we used GW4869 to treat MSC^NS‐Exo^ for inhibiting the ability of releasing exosomes (Figure S7). We established co‐culture system and collected the conditioned medium from MSC^NS‐Exo^ to treat ECs and examined the changes on the abilities of proliferation, migration and tube formation. The results showed that the functions of MSC^NS‐Exo^ were blocked after the application of GW4869 in promoting angiogenesis, indicating the exosomes released by the educated MSCs play a key role in therapeutic process (Figure S8). Moreover, we explored the detail mechanisms underlying angiogenesis activated by exosomes. Since vascular endothelial growth factor (VEGF) signalling is critical for regulating angiogenesis, we examined the expression of several important proteins in this pathway.^29^, ^30^, ^31^ We found the protein expression levels of p‐AKT, p‐eNOS in ECs increased after application of exosomes, especially in NM‐Exo group (Figure 6G, H). These data revealed that the exosomes released from educated MSCs stimulated angiogenesis and involved in activation of AKT‐mediated VEGF signalling pathway.

**FIGURE 7:**
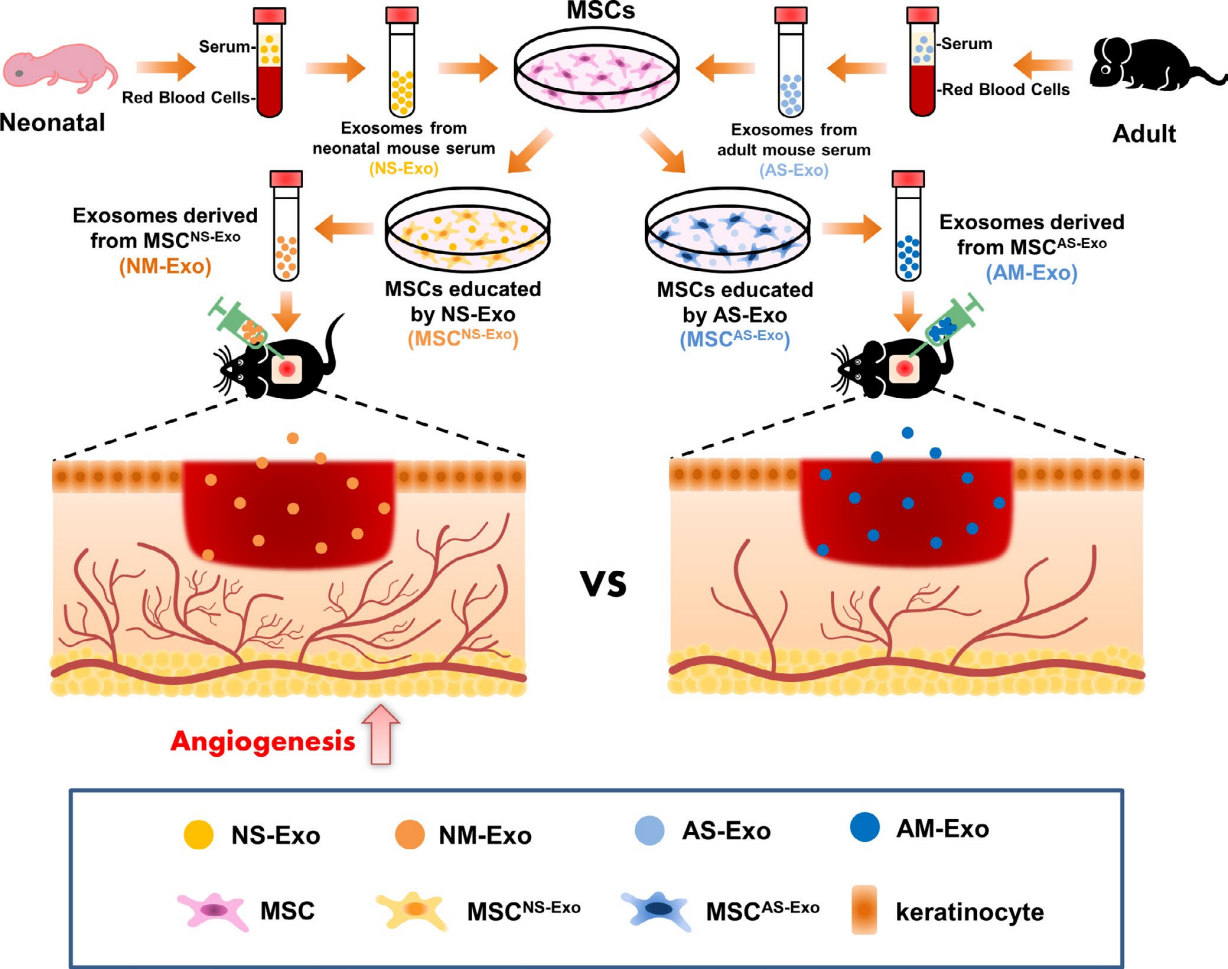
Schema for exosomes derived from the educated MSCs promoted angiogenesis in cutaneous wound healing via regulating the functions of endothelial cell

In this study, we found that the MSCs which educated by exosomes from neonatal serum could possess advances in the therapeutic effect on cutaneous wound healing. Meanwhile, the exosomes derived from the MSCs educated by exosomes from neonatal serum previously had the better ability to regulate the functions of endothelial cells which are important for the angiogenesis in wound healing process (Figure 7).

## 4 DISCUSSION

In current study, our results demonstrated that the exosomes from neonatal serum could enhance the biological functions of mesenchymal stem cells (MSCs). In addition, we found that the extracted exosomes from educated MSCs also had more powerful therapeutic effect on cutaneous wound healing which promoted the angiogenesis via regulating the functions of endothelial cells. Therefore, our study not only proved the lasting effect of exosomes from neonatal serum on enhancing the functions of MSCs but also provided a promising cell‐free therapeutic strategy for skin injuries.

Skin plays an important role in the physical defence system of human beings, so that the therapies for the skin injuries are always highly focused. MSCs are the kind of stem cells that possess the capacity of self‐renewal and multi‐directional differentiation,^32^ which can be isolated from multiple tissues like bone marrow, umbilical cord, adipose tissue and so on.^33^ Previous studies have demonstrated that MSC therapy had the positive effects on cutaneous wound healing in different models.^34^, ^35^, ^36^ However, pre‐clinical studies have demonstrated that cell survival and retention rate are closely related to the outcome of MSC‐mediated therapy.^37^ These studies demonstrated that although MSCs can undergo differentiation into various somatic cells under defined conditions in vitro, they rarely transform into target cells after transplantation. Meanwhile, the transplanted MSCs undergo apoptosis or senescence in response to the harsh microenvironment. An obstacle facing MSC‐based therapy is the limited number of functional stem cells available after transplantation due to the harsh microenvironment, like anoikis and inflammation induced by damaged tissues or organs.^38^, ^39^ Therefore, improving cell function is pertinent to promote the therapeutic efficacy of MSC therapy.

To overcome this limitation, various strategies for enhancing the functions of MSCs have been adopted which have shown promising results.^40^ These approaches can be categorized as pre‐treatment with growth factors or cytokines, pre‐conditioning such as hypoxia and genetic modifications to strengthen the functions of MSCs.^41^, ^42^, ^43^, ^44^, ^45^, ^46^ Extracellular vesicles (EVs) have attracted considerable attention because they play the important roles in cell‐to‐cell communications in physiological and pathological conditions.^47^ Exosomes serve as a population of EVs, which shown as the important regulators of cell functions.^14^, ^15^ Recent studies indicate that tumour‐derived exosomes are important tumorigenesis mediators capable of educating stem cells for neoplastic transformation and tumour metastasis, and stem cell‐derived exosomes are able to promote cancer cell functions.^48^, ^49^ As a kind of extracellular vesicles compared to chemical compounds, exosomes derived from the living cells can be more biocompatible. The cargos of exosomes are abundant and stable, so the application of exosomes is more efficient than cytokines. Compared to genetic modifications or physical methods, this protocol is easier to carry out and safer. For instance, although genetic modification by overexpression or gene knockout was demonstrated to improve the therapeutic efficacy of MSCs, the use of techniques involving viral or non‐viral modification prompts safety concerns, particularly that the modification may promote the tumorigenesis of MSCs. Thus, it is necessary to guarantee the safety of the modified MSCs for potential clinical applications in regenerative medicine.^6^, ^16^, ^50^

Moreover, it is known that the regenerative ability of mammals decreases with ageing.^51^ For example, the adult mammalian heart possesses limited potential for repair and regeneration, while in neonatal mice, the heart can regenerate fully without scarring following injury.^52^ In the treatment of myocardial infarction, the researchers found that MSCs from younger donors are more efficacious than those from older donors.^53^ Previous studies suggest that the circulating environment of young animals has the ability to promote tissue regeneration and reverse age‐related impairments, and the molecular signalling pathways which critical to the activation of tissue‐specific progenitor cells can be modulated by circulating factors.^54^, ^55^ Utilizing a parabiosis model between young and old mice, researchers have demonstrated that the high levels of GDF11 in young blood are beneficial to stem cell functions and skeletal muscle regeneration.^56^ However, the role of circulating exosomes in young blood has not been investigated. At present, studies on circulating exosomes mainly focus on their role as biomarkers for disease diagnosis or as the delivery vehicles for therapy.^57^, ^58^, ^59^ Recent studies have revealed that circulating exosomes reflect the physiological conditions of the body and can be stimulated by exercise.^60^ Hence, the exercise‐induced circulating exosomes mediate beneficial biological effects on tissues, such as cardioprotective effects, demonstrating that circulating exosomes enhance physiological functions of the body.^61^ Therefore, our study considered the potential role of circulating serum exosomes in neonatal blood and used them to pre‐treat MSCs which have not been reported yet. In this study, we compared the differences between the exosomes from neonatal and adult serum. Then, we investigated the biological effect of two exosomes on MSCs and compared the therapeutic ability between these two educated MSCs. We found that the MSCs educated by exosomes from neonatal serum had a better effect on wound healing process. This finding further underlines the importance of exosomes from young individuals in maintenance of stem cell functions and shows the good effect on pre‐treatment of the exosomes, which enriches our understanding of the role of exosomes in maintaining the cell and tissue homeostasis.

In recent years, the paracrine pathway has been recognized, and exosomes have been demonstrated to be the key factor mediating the functions of MSCs. Many studies have confirmed the therapeutic effect of exosomes as a cell‐free therapy in multiple tissue injury models.^16^ MSC transplantation has some disadvantages and risks, for example, the microenvironment of damaged tissue is not conducive to the survival of MSCs, which resulting in a low retention rate and affecting the therapeutic effect. Exosomes are rapidly emerging as a promising therapeutic platform for cell‐free application with the advantages of immune privilege, signalling transfer and efficiency for endocytosis.^62^ Recent studies indicated that the therapeutic effects of MSC administration that happened via paracrine mechanism and exosomes can be directly used to enhance the cutaneous wound healing by promoting angiogenesis.^63^ Exosomes are smaller, less complex, less immunogenic and more biocompatible than cells, so that application of exosomes is safer. Meanwhile, the quality of exosomes is more controllable, and the cargos of exosomes are more stable, so that exosomes are more convenient to product, store and transport. Unlike living cells, there is no need to worry about the survival and retention rate.^50^ In our study, we found that the exosomes released by educated MSCs had the powerful ability to treat the cutaneous wound healing, which is consistent with the effect of MSC therapy. Moreover, we revealed that exosomes released from MSCs which educated by exosomes from neonatal serum before had a more outstanding performance through enhancing the functions of endothelial cell via regulating the related signalling pathway.

In conclusion, our study shed light on the efficacy of exosomes in tissue repair, which widens the research scope of extracellular vesicles. Meanwhile, our study provides new evidence to expand the possibility of pre‐treatment of MSCs as the strategy. In addition, the underlying mechanism through which the upgraded exosomes released by educated MSCs could promote angiogenesis that can be applied in tissue engineering and regenerative medicine by regulating the biological properties of implanted MSCs.

## CONFLICT OF INTEREST

The authors have no conflicts of interest to declare.

## AUTHOR CONTRIBUTION

Xinyu Qiu, Jin Liu and Chenxi Zheng contributed equally to the study design, manuscript preparation and data collection. Yuting Su, Lili Bao and Bin Zhu made contributions to design, data acquisition, and drafted the manuscript. Siying Liu, Lulu Wang and Xiao Wang contributed to animal experiment, data analysis and drafted the manuscript. Yirong Wang, Wanmin Zhao and Jun Zhou provided the data acquisition and critically revised the manuscript. Zhihong Deng contributed to the study conception and critically revised the manuscript. Shiyu Liu and Yan Jin supervised the research, oversaw the collection of results and data interpretation and critically revised the manuscript. All authors approved the final manuscript as submitted and agree to be accountable for all aspects of the work.

## Supporting information

Supplementary Material

## Data Availability

The data sets used and/or analysed during the current study are available from the corresponding author on reasonable request.
