# Supplementary material for: Exosomes released from educated mesenchymal stem cells accelerate cutaneous wound healing via promoting angiogenesis
Source: Cell Prolif. 2020 Jul 1;53(8):e12830. doi: 10.1111/cpr.12830 (PMC7445410; doi:10.1111/cpr.12830)
Supplement: Supplementary file 1 — Supplementary Material [file CPR-53-e12830-s001.docx]

**Supporting Information**

**Exosomes released from educated mesenchymal stem cells accelerate cutaneous wound healing via promoting angiogenesis**

**Xinyu Qiu ^1,^**^✟^**, Jin Liu ^1,3,^**^✟^**, Chenxi Zheng ^1,2,^**^✟^**, Yuting Su ^4^, Lili Bao ^2^, Bin Zhu ^1,5^, Siying Liu ^6^, Lulu Wang ^7^, Xiao Wang ^8^, Yirong Wang ^8^, Wanmin Zhao ^1,2^, Jun Zhou ^1^, Zhihong Deng ^1^, Shiyu Liu ^1,^* and Yan Jin ^1,^***

^1^ State Key Laboratory of Military Stomatology& National Clinical Research Center for Oral Diseases & Shaanxi International Joint Research Center for Oral Diseases，Center for Tissue Engineering, School of Stomatology, The Fourth Military Medical University, Xi’an, Shaanxi 710032, China;

^2^ Xi’an Institute of Tissue Engineering and Regenerative Medicine, Xi’an, Shaanxi 710032, China;

^3^ State Key Laboratory of Military Stomatology & National Clinical Research Center for Oral Diseases & Shaanxi Key Laboratory of Stomatology, Department of Prosthodontics, School of Stomatology, The Fourth Military Medical University, Xi'an, Shaanxi, 710032, China;

^4^ Department of aerospace, The Fourth Military Medical University, Xi’an, Shaanxi 710032, China;

^5^ Department of Stomatology, The General Hospital of Tibet Military Region, Lhasa, Tibet, 850007, China;

^6^ State Key Laboratory of Military Stomatology & National Clinical Research Center for Oral Diseases & Shaanxi Clinical Research Center for Oral Diseases, Department of Orthodontics, School of Stomatology, The Fourth Military Medical University, Xi’an, Shaanxi, 710032, China;

^7^ State Key Laboratory of Military Stomatology & National Clinical Research Center for Oral Diseases and Shaanxi Clinical Research Center for Oral Diseases, Department of Pediatric Dentistry, School of Stomatology, The Fourth Military Medical University, Xi'an, 710032, China;

^8^ State Key Laboratory of Military Stomatology & National Clinical Research Center for Oral Diseases & Shaanxi Key Laboratory of Oral Diseases, Department of Operative Dentistry and Endodontics, School of Stomatology, The Fourth Military Medical University, Xi'an, China.

***** Correspondence: yanjin@fmmu.edu.cn (Y.J.) liushiyu@vip.163.com (S.Y.L.)

✟ These authors contributed equally to this work.


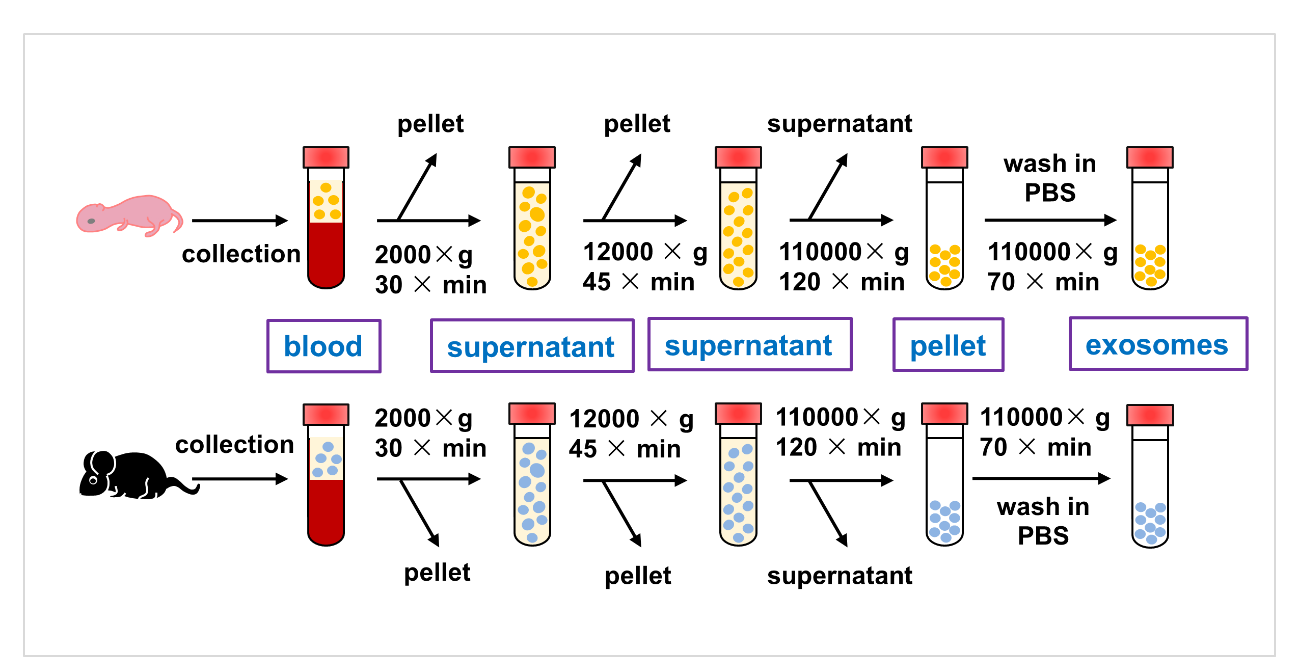


**Figure S1.** The schematic graph shows the protocol of isolating the exosomes from the serum of neonatal and adult mice.


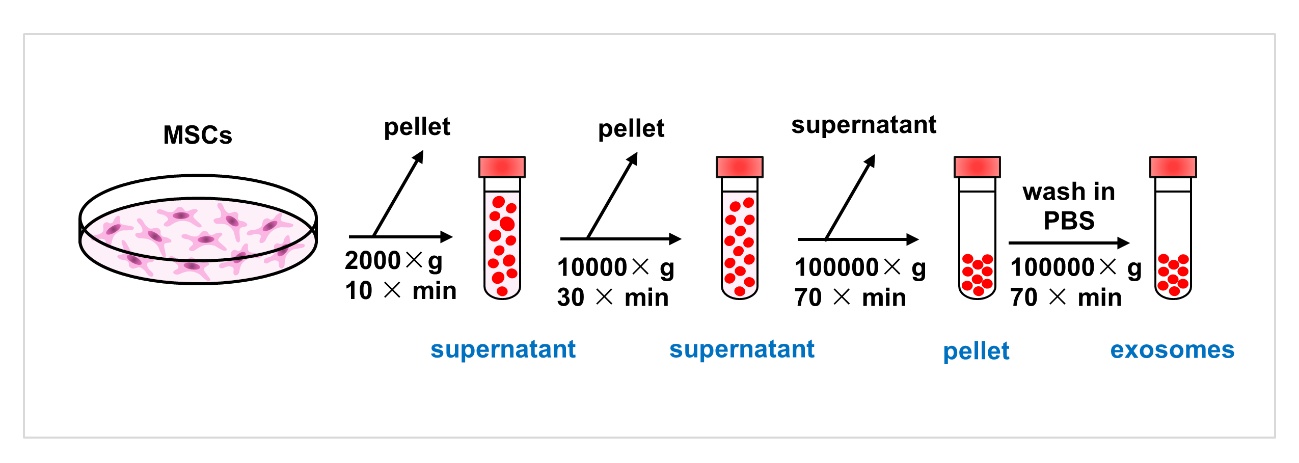


**Figure S2.** The schematic graph shows the protocol of isolating the exosomes from the conditioned medium of the educated MSCs.


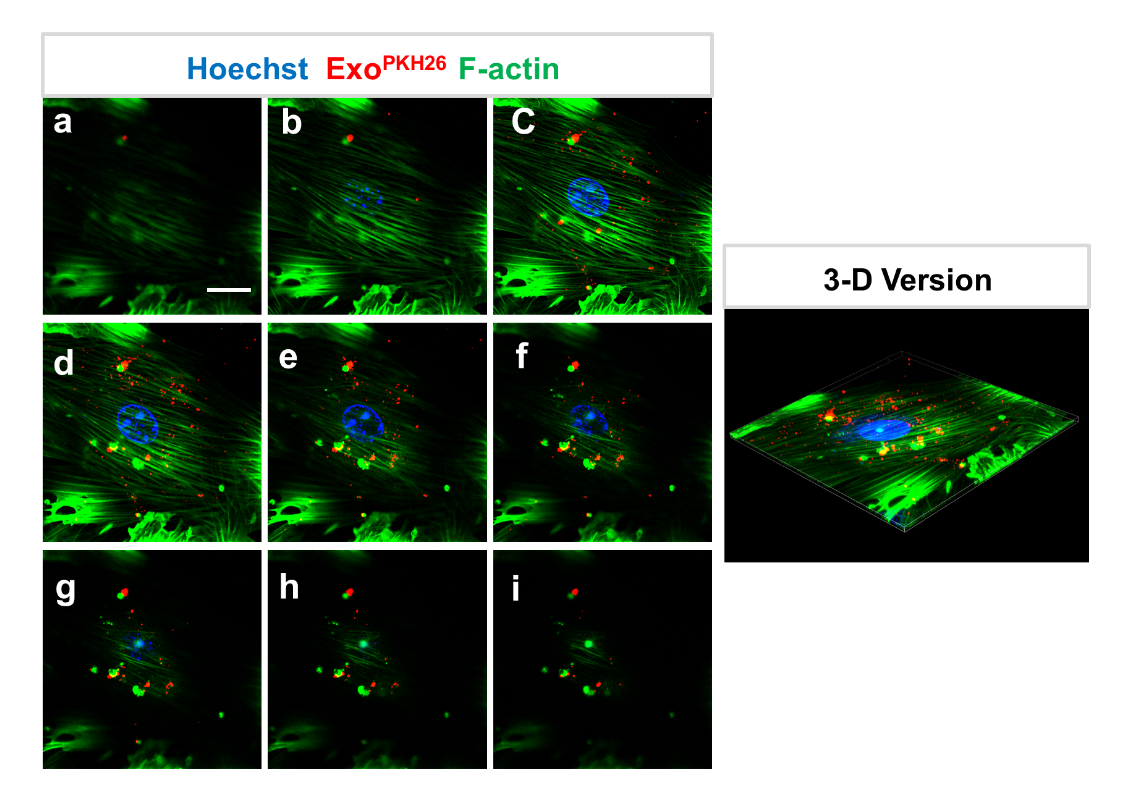


**Figure S3.** Confocal microscope images of mesenchymal stem cells after co-culture with exosomes focusing on various positions in *Z*-axis. Scale bar, 20 μm.


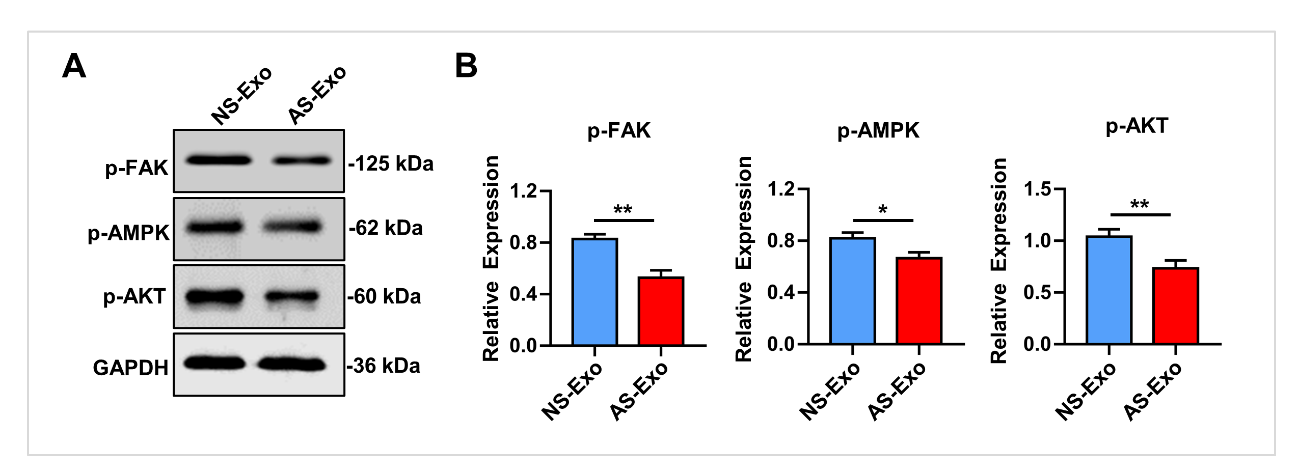


**Figure S4.** (A-B) The protein expression levels of p-FAK, p-AMPK and p-AKT were detected by western blot in the two kinds of exosomes which were extracted from serum of neonatal and adult mice. The results were quantified by ImageJ software. n=3 per group. Data are shown as mean ± SD; **P* < 0.05; ***P* < 0.01. NS-Exo, neonatal serum exosomes; AS-Exo, adult serum exosomes.


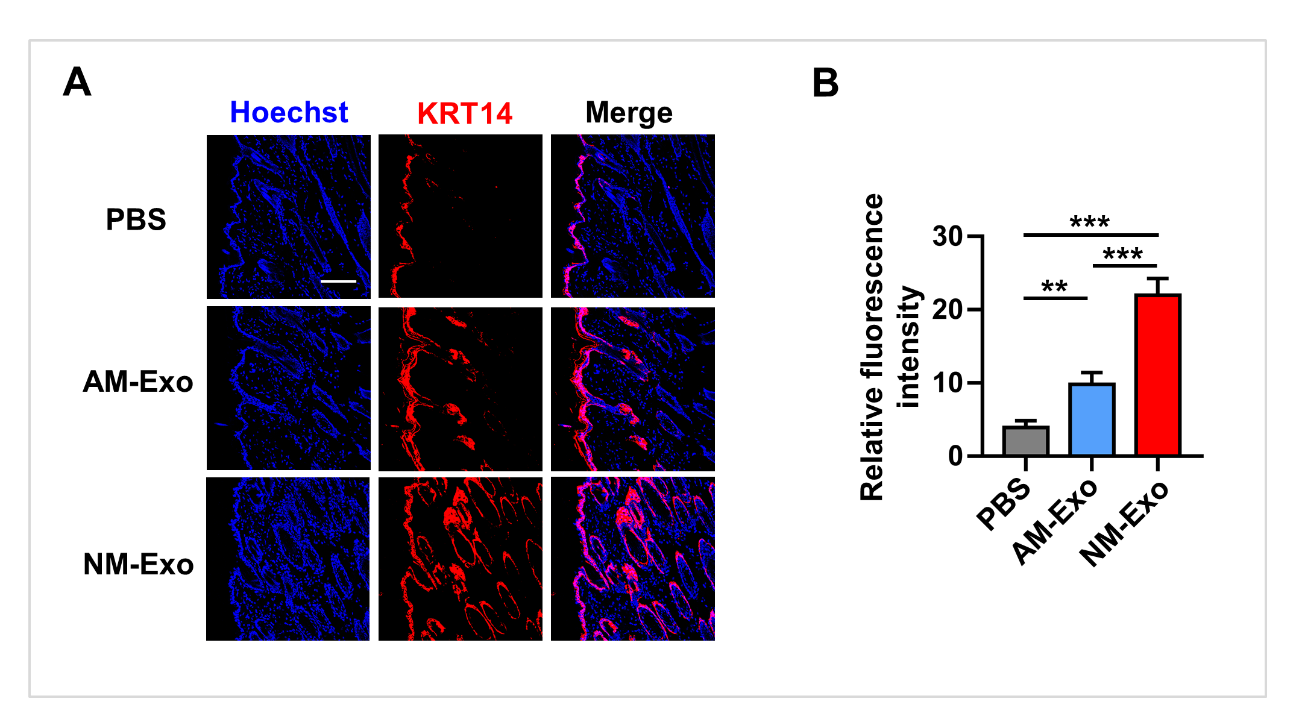


**Figure S5.** Exosomes promoted the re-epithelization during cutaneous wound healing process *in vivo*. (A-B) Representative images and quantitative analysis of the KRT14 expression in the skin tissue samples in different groups. Scale bar, 100 μm. n=3 per group. Data are shown as mean ± SD; ***P* < 0.01, ****P* < 0.001. PBS, phosphate buffer saline; NM-Exo, exosomes derived from MSC^NS-Exo^; AM-Exo, exosomes derived from MSC^AS-Exo^.


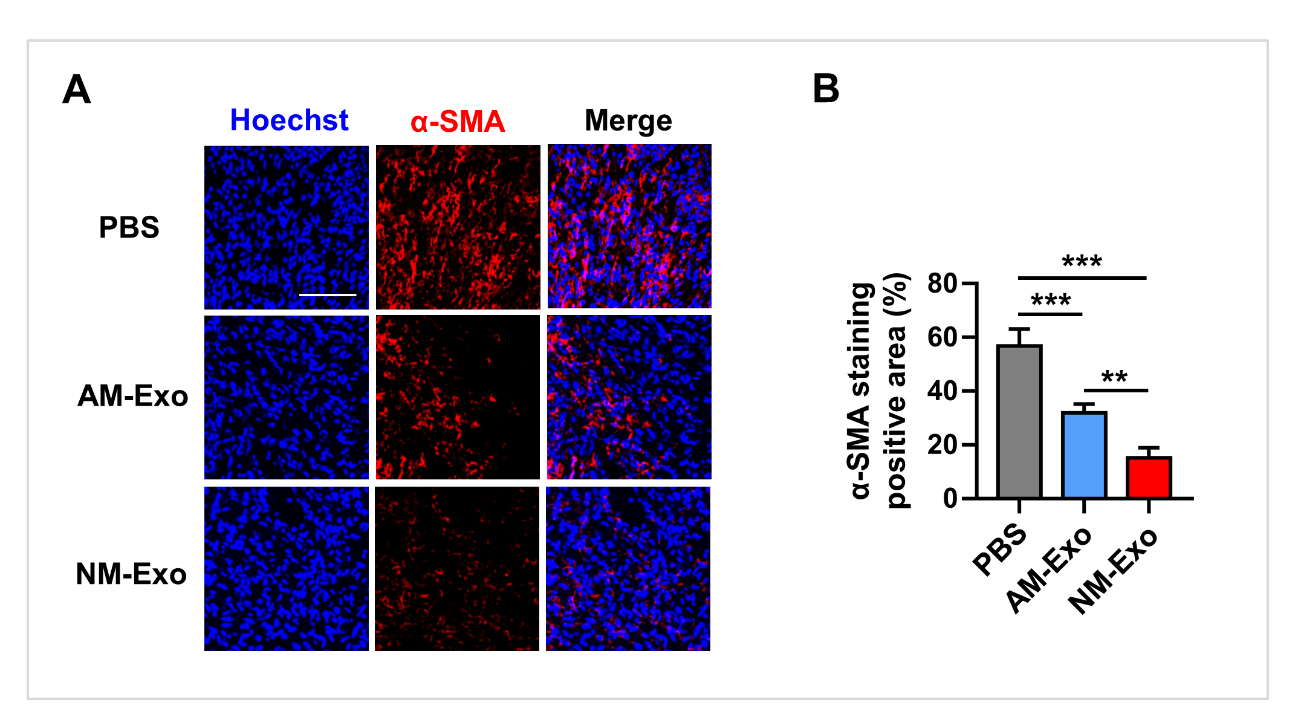


**Figure S6.** Exosomes inhibited the fibrosis during cutaneous wound healing process *in vivo*. (A-B) Representative images and quantitative analysis of the α-SMA expression in the skin tissue samples in different groups. Scale bar, 100 μm. n=3 per group. Data are shown as mean ± SD; ***P* < 0.01, ****P* < 0.001. PBS, phosphate buffer saline; NM-Exo, exosomes derived from MSC^NS-Exo^; AM-Exo, exosomes derived from MSC^AS-Exo^.


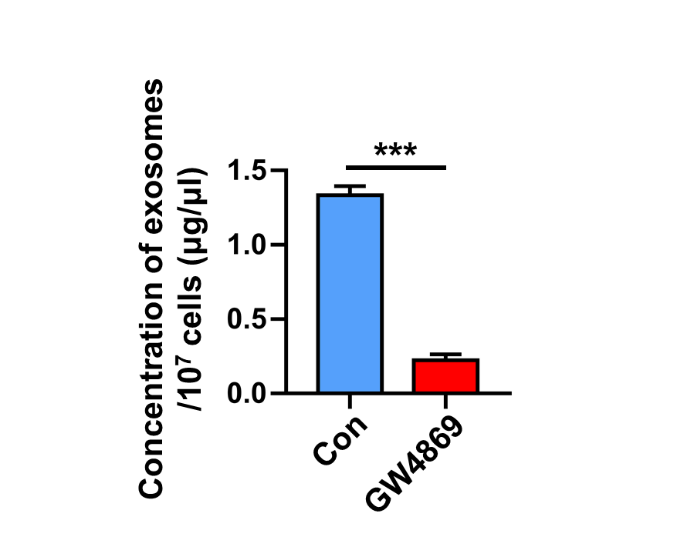


**Figure S7.** GW4869 inhibited exosome secretion in MSCs *in vitro*. The protein concentration of released exosomes was detected by BCA assay with or without using GW4869. n=3 per group. Data are shown as mean ± SD, ****P* < 0.001.


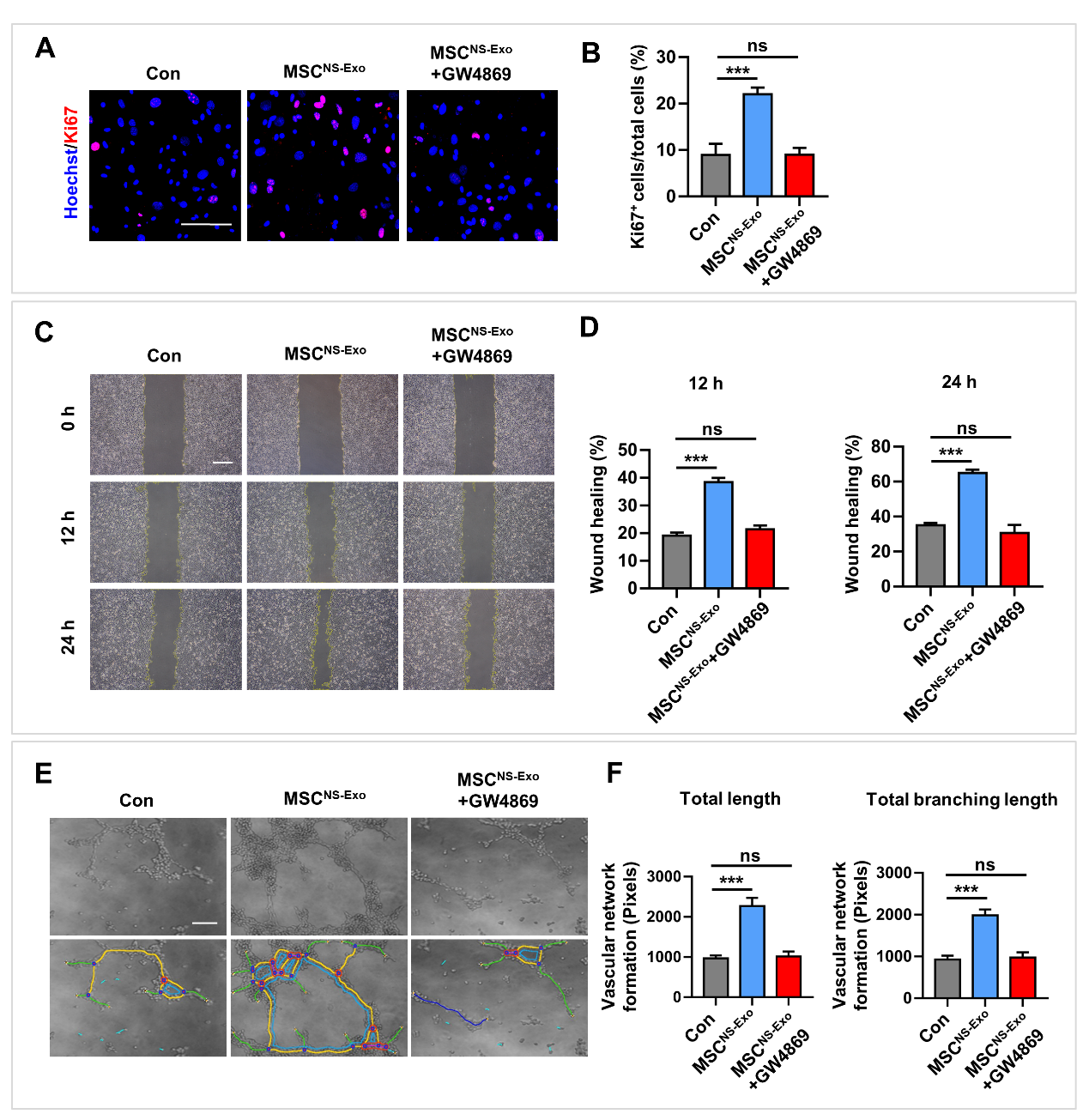


**Figure S8.** GW4869 blocked the pro-angiogenic functions of MSC^NS-Exo^ on endothelial cells *in vitro*. (A-B) Proliferation was detected by Ki67 staining and quantified by the positive-stained percentage. Scale bar, 100 μm. (C-D) Representative images and quantification of scratch assay examining the migration ability of endothelial cells which co-cultured with MSC^NS-Exo^ or MSC^NS-Exo^+GW4869. Scale bar, 500 μm. (E-F) Tube formation capacity on the Matrigel and the images were analyzed by ImageJ software. Scale bar, 20 μm. n=3 per group. Data are shown as mean ± SD; ns, not significant; ****P* < 0.001. Con, control; MSC^NS-Exo^, MSCs educated by exosomes from neonatal serum; MSC^NS-Exo^+GW4869, MSCs educated by exosomes from neonatal serum which pre-treated by GW4869.
